# Cholesterol Crystallization Tests the Limits of Microscopic Reversibility for Molecular Pathways of Growth and Dissolution

**DOI:** 10.1021/acscentsci.5c02371

**Published:** 2026-04-24

**Authors:** Dipayan Chakraborty, Muhammad Osman Khalid, Peter G. Vekilov, Jeffrey D. Rimer

**Affiliations:** † William A. Brookshire Department of Chemical and Biomolecular Engineering, 14743 University of Houston, 4226 Martin Luther King Blvd., Houston, Texas 77204-4004, United States; ‡ Welch Center for Advanced Bioactive Materials Crystallization, 14743 University of Houston, 4226 Martin Luther King Blvd., Houston, Texas 77204-4004, United States; § Department of Chemistry, 14743 University of Houston, 3585 Cullen Blvd., Houston, Texas 77204-5003, United States

## Abstract

A subject often debated in the field of crystallization is whether opposing processes of growth and dissolution abide by microscopic reversibility under conditions far from equilibrium. This is particularly relevant for nonclassical growth pathways such as crystallization by particle attachment (CPA), which is an irreversible process owing to the unique sequence of steps involving precursor attachment to crystal surfaces followed by their disorder-to-order transitions as they integrate into the underlying crystal. The reverse process of crystal dissolution is frequently associated with the retraction of unfinished layers, which is the opposite of classical growth and distinctly different than CPA. Here, we selected cholesterol as a model system based on its prevalence in physiological environments that can switch between conditions of supersaturation (crystal growth) and undersaturation (crystal dissolution). We leverage our recent observations that cholesterol nucleation and growth occur by nonclassical and classical pathways, respectively. Our findings reveal the limits of microscopic reversibility when working in moderately undersaturated solutions where dissolution occurs by a unique nonclassical pathway involving the formation of cholesterol-rich clusters on crystal surfaces. Attempts to regenerate crystal surface growth after clusters are formed reveal they have an irreversible effect on crystallization in ways that potentially inhibit pathological cholesterol precipitation.

## Graphical abstract

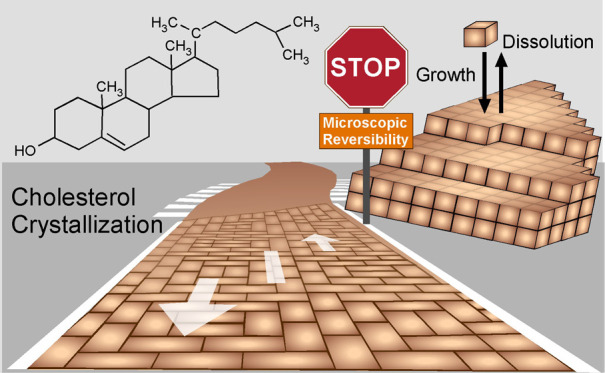

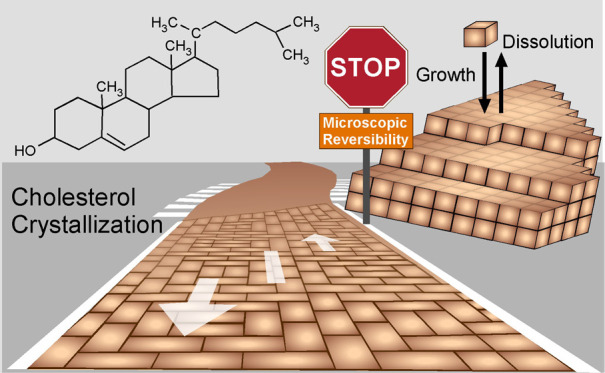

## Introduction

Cholesterol monohydrate is the predominant crystalline form of cholesterol found in pathological deposits such as gallstones and atherosclerotic plaques.^1–3^ In the human body, cholesterol monohydrate adopts a triclinic crystal lattice, in which cholesterol molecules are stabilized by hydrogen bonds between their hydroxyl groups and water.^4^ Cholesterol monohydrate crystals constitute up to 80% of the mass of gallstones,^5^ which form through a complex process driven by cholesterol supersaturation in bile.^6,7^ Cholesterol monohydrate crystals also play a significant role in cardiovascular disease, where they contribute to plaque development, inflammation, and tissue damage.^8–10^ Cholesterol’s poor water solubility promotes its adhesion to the hydrophobic surfaces of arterial walls.^11–15^ The characteristic plate-like morphology of cholesterol monohydrate crystals influences their aggregation and deposition in biological environments, exacerbating disease progression via mechanical obstruction.^16–18^ The stability and slow dissolution of cholesterol monohydrate in physiological fluids present challenges in therapeutic interventions, making it crucial to understand the fundamental processes leading to its formation and dissolution.

Surgical removal of the gallbladder remains the most common and effective treatment for gallstone disease due to the limited effectiveness of other therapies.^19^ Gallstones affect over 20 million Americans, resulting in 2.2 million hospitalizations each year at an estimated cost of $6.5 billion.^20,21^ Therefore, there is an increased need for the development and administration of safe and noninvasive treatments. Dissolution therapy is a promising alternative to surgical procedures but is seldom preferred due to lengthy treatment times and its inability to suppress gallstone recurrence.^22,23^ Recently, bile salts have been used to dissolve cholesterol gallstones successfully. Examples include chenodeoxycholate and ursodeoxycholate that have been used to desaturate cholesterol in the bile and induce dissolution;^24,25^ however, these therapies have only been successful for a fraction of patients.

Dissolution of pathological crystal deposits and its potential therapeutic applications have been investigated in literature. For instance, studies elucidating the molecular mechanisms of dissolution for crystals implicated in kidney stone disease provide useful insights toward alleviating and preventing urolithiasis.^26–29^ By contrast, dissolution of cholesterol monohydrate crystals is a seldom reported subject. Most dissolution studies on cholesterol have focused on bulk dissolution of gallstones with little to no emphasis on the surface phenomena or molecular mechanisms of dissolution.^30–32^ Swift and co-workers have examined the dissolution of cholesterol by atomic force microscopy (AFM) in ethanol/water mixtures in the absence and presence of bile salts. Their results revealed a mechanism of dissolution involving a combination of etch pit formation and the retraction of steps, composed of cholesterol bilayers.^33,34^ In a previous study,^35^ we showed that cholesterol crystallization in the presence of select alcohols can result in the formation of cholesterol solvates, such as cholesterol hemiethanolate and monohydrate that form in aqueous solutions with ethanol and isopropyl alcohol (IPA), respectively. One interesting outcome of using water/ethanol mixtures is the observation of mesoscopic cholesterol-rich clusters,^36^ which are expected to host nonclassical crystal nucleation, but were not seen to partake in crystal growth.

In this study, we examine molecular mechanisms of dissolution using both cholesterol monohydrate and hemiethanolate crystals in two alcohol/water solvents under varying degrees of undersaturation and alcohol/water ratios. We observe that undersaturated solutions near equilibrium induce a combination of layer retraction and etch pit formation. As the degree of undersaturation is increased, taking the system further from equilibrium, there is an unexpected switch to nonclassical dissolution that deviates from the microscopic reversibility of classical layered growth. This nonclassical pathway is associated with the formation of protrusions that resemble cholesterol-rich clusters in bulk solution that violate the reversibility of cholesterol surface growth dynamics. To our knowledge, this is the first observation of this pathway occurring for any material in a regime of crystal dissolution.

## Results and Discussion

### Microscopic Reversibility of Crystal Growth and Dissolution

Classical growth of crystal surfaces occurs by the generation and spreading of layers via the attachment of monomers. New layers may originate on dislocations or two-dimensional nuclei and their advancement involves the incorporation of solute ions/molecules either directly from solution or after diffusion along the terraces separating adjacent layer edges, also referred to as steps (Figure 1A).^37–39^ In the case of cholesterol monohydrate and hemiethanolate crystallization, we previously showed that surfaces grow by a classical mechanism where unfinished layers advance from screw dislocations by the addition of solute via a surface diffusion pathway.^35,36^ Atomic force microscopy (AFM) images of cholesterol monohydrate (001) surfaces, collected *in situ* in a supersaturated solution during growth, reveal a distribution of single layers (Figure 1B, white arrow), and multilayer formations of various heights (Figure 1B, yellow arrow and white line). The surface diffusion mechanism creates a disparity in step velocity with single layers growing much faster than multilayers, and bunches of multiple steps showing no signs of appreciable advancement. At equilibrium, the rates of solute attachment (growth or forward direction) and detachment (dissolution or backward direction) are equal. This condition of microscopic reversibility establishes the equilibrium constant for crystallization, *K*_*i*_ *= k*_*i,f*_/*k*_*i,b*_, where *k*_*i*_ are the rates of reaction in the forward (*k*_*i,f*_) and backward (*k*_*i,b*_) directions.^40^

**Figure 1:**
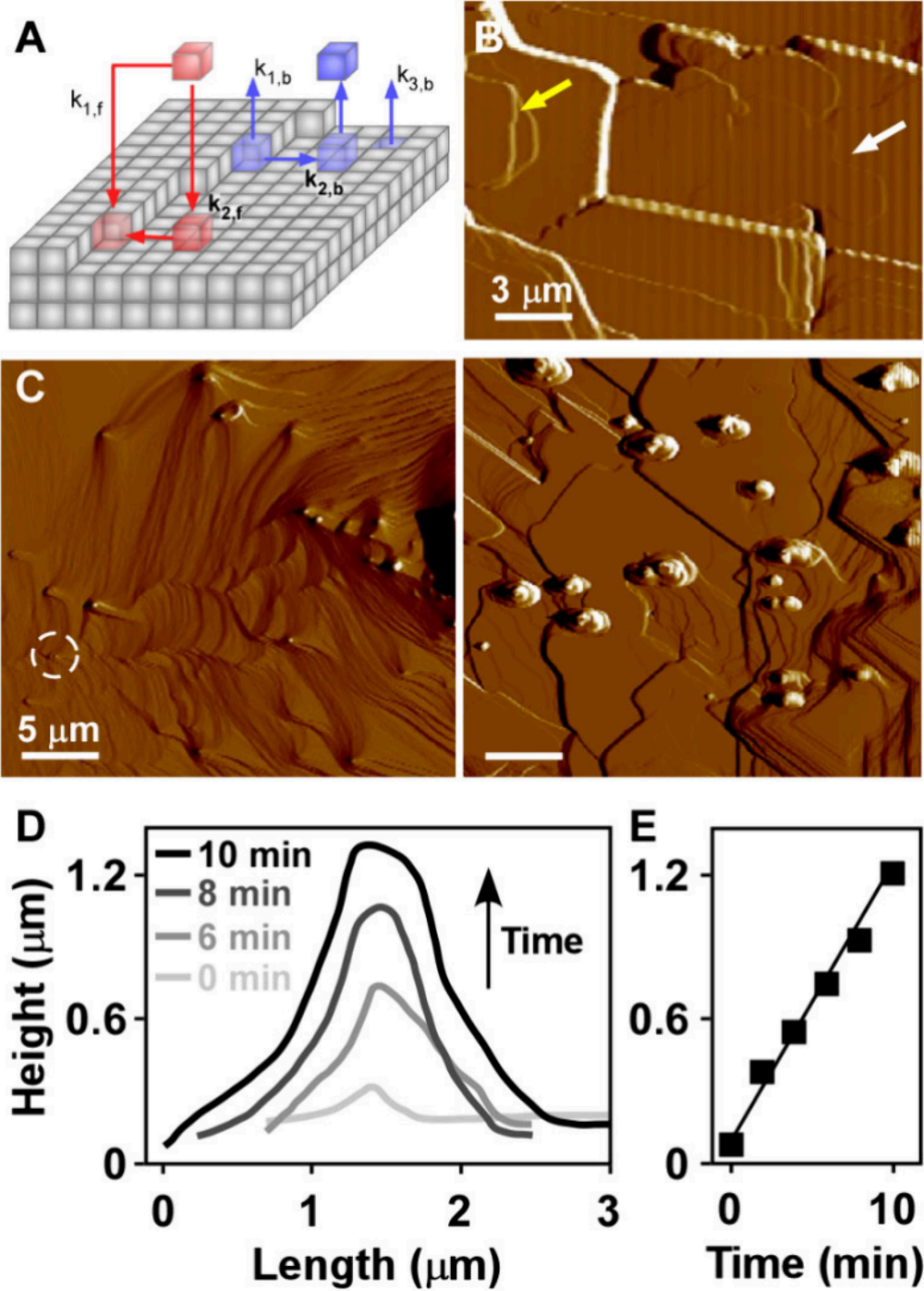
Phenomenology of cholesterol dissolution. (A) Idealized crystal schematic showing pathways with corresponding rates (*k*_*i*_) for growth (red) by direct addition to kink sites (*k*_*1,f*_) and surface diffusion (*k*_*2,f*_), and dissolution (blue) by three possible pathways: direct detachment (*k*_*1,b*_), the reverse of surface diffusion (*k*_*2,b*_), and the generation of surface vacancies (*k*_*3,b*_). (B) AFM image showing a representative (001) surface of a triclinic cholesterol monohydrate crystal during *in situ* growth in a water/IPA solution (50% H_2_O by volume) with 1.45 mM cholesterol (where *C*_eq_ = 1.32 mM)^35^ and a flow rate of 60 mL h^–1^. (C) AFM images of cholesterol monohydrate (001) surfaces dissolving in a water/IPA solvent (35% H_2_O by volume) using a flow rate of 10 mL h^–1^. The image on the left was extracted from Movie S1 (0 min), and the image on the right was taken from a separate experiment showing a high density of protrusions. (D) Height profile versus length for the protrusion highlighted in panel (C) (dashed circle). (E) Corresponding evolution of maximum protrusion height with time.

It is often assumed that microscopic reversibility for any reaction implies the pathways of forward and backward directions follow the same series of steps, but in reverse order.^41,42^ The applicability of this principle to crystal growth has been found to remain valid when the system is relatively close to equilibrium (i.e., the solute concentration is slightly below the crystal solubility, *C*_eq_).^43,44^ Here, we test whether cholesterol dissolution obeys microscopic reversibility and assess how far from equilibrium this condition is maintained. Crystal dissolution can involve a series of pathways such as layer retraction and etch pit formation.^43,45^ Since it has already been established that the surface growth of cholesterol crystals is not dominated by direct solute incorporation (Figure 1A, *k*_*1,f*_) but rather involves surface diffusion (Figure 1A, *k*_*2,f*_), it would be expected on the basis of microscopic reversibility that surface dissolution occurs by the reverse process (Figure 1A, *k*_*2,b*_) rather than direct detachment of solute from kink sites to the bulk solution (Figure 1A, *k*_*1,b*_). Surface diffusion during growth can be confirmed by correlations between step advancement and interstep distance.^46^ One pathway that would indicate a deviation from microscopic reversibility is etch pit formation (Figure 1A, *k*_*3,b*_). Etch pits are initiated by the generation of terrace vacancies, which do not exist during growth and thus, monomer addition to terrace vacancies is not a common pathway of surface growth. Herein, we explore cholesterol dissolution over a range of solution conditions, both near and far from equilibrium, to ascertain what pathways are dominant during dissolution.

### Interfacial Phase Transformation during Cholesterol Dissolution

Cholesterol is an amphiphilic molecule that crystallizes in mixed organic/water solvents. We previously showed^35^ that triclinic cholesterol monohydrate crystals can be prepared with select organics, such as isopropyl alcohol (IPA), similar to prior studies reporting crystallization in lipids.^47–50^ We also showed that the selection of low molecular weight alcohols, such as ethanol, as the organic solvent lead to the formation of cholesterol solvates (e.g., cholesterol hemiethanolate).^36^ Solvent occluded within the crystal structure can be easily exchanged with the surrounding medium. To confirm this, we placed different cholesterol solvate crystals in solutions of either pure alcohol or pure deionized (DI) water. For experiments where cholesterol monohydrate crystals were introduced into pure ethanol, we observed a complete conversion to cholesterol hemiethanolate (Figure S1A); the crystals did not dissolve during this conversion indicating that the ethanol invaded the lattice. A similar phenomenon was observed for cholesterol hemiethanolate, which converted to cholesterol monohydrate when placed in pure DI water (Figure S1A). When both cholesterol solvate crystals were exposed to air at ambient conditions, we observed that monohydrate crystals are stable whereas the ethanol occluded within hemiethanolate crystals evaporated over several hours to produce anhydrous cholesterol crystals (Figure S1B).

We first measured the dissolution of cholesterol monohydrate using *in situ* AFM to capture the dynamics of a (001) surface in contact with a 35% water/65% IPA mixture in which no cholesterol was added. The unfinished layers retract (Figure 1C, Movie S1), which is the reverse process of growth in a supersaturated solution. In addition, we observe the formation of a new phase that emerges as protrusions (Figure 1C, dashed circle) and continues to grow in size as the underlying layers dissolve (Figure 1D). These protrusions grow by a pathway resembling nonclassical crystallization involving either precursor attachment^51^ or 3-dimensional growth of islands by the addition of soluble species.^52^ Tracking the exact morphology of these protrusions with the AFM cantilever is challenging owing to their large size (0.3–1.2 μm in height). We were able to monitor changes in the maximum height with time, which exhibits a linear behavior (Figure 1E) with a rate of 2 nm s^–1^ (out-of-plane). This is nearly 7-times slower than layer advancement (in-plane) during cholesterol monohydrate surface growth at low supersaturation (C/C_eq_ = 1.02).^35^

Attempts to measure the velocity of step retraction were difficult due to the fast rate of layer dissolution in cholesterol-free solvent. We performed similar AFM measurements for a cholesterol hemiethanolate (010) surface in a 35% water/ethanol solution, which revealed similar nonclassical formation of protrusions (Figure S2) in higher numbers and unfinished layers that dissolved at much slower rates (*vide infra*), making them easier to track *in situ*. Therefore, in the remainder of this study we focus on the dissolution of cholesterol hemiethanolate crystals as a model system. One of the unique aspects of this system is the presence of cholesterol-rich clusters (ca. 100 nm) that form in water/ethanol solutions over a range of cholesterol concentrations,^36^ spanning from undersaturated to supersaturated conditions. These clusters are more difficult to study in water/IPA mixtures used to prepare cholesterol monohydrate owing to the formation of alcohol/water emulsions. Here we hypothesize that the nonclassical protrusions formed at the crystal–solvent interface during cholesterol dissolution are a new phase resembling the clusters in bulk solution that are believed to participate in nucleation but are spectators during surface growth.

### Effect of Water Content on Interfacial Phase Transformation

We previously reported that the equilibrium concentration (C_eq_) of cholesterol in water/ethanol mixtures decreases with increased water content.^35^ In the same study we also showed that higher water content resulted in a significant increase in the number of cholesterol-rich clusters and only a slight increase in their average size. Here, we assessed crystal dissolution in cholesterol-free solvents in which the water content varied within the range of 20 to 60% H_2_O (v/v). At the lowest water content (i.e., at which C_eq_ is the highest and the relative undersaturation (C – C_eq_) = −C_eq_ is the highest), the rate of dissolution is much faster than that at higher water content. The generation and growth of surface protrusions under different solvent compositions was tracked *in situ* by AFM. At the lowest water content (20%), dissolution results in a large number of protrusions within a relatively short period of imaging (Figure 2A). As the water content is increased to 35% (Figure 2B) and then 60% (Figure 2C), there is a progressive reduction in the number of protrusions. Time-resolved analysis of AFM images during these measurements (Figure S3) reveal three distinct dynamic modes of surface dissolution: (i) etch-pit formation, (ii) layer retraction, and (iii) formation and growth of protrusions. At the lowest water content (Figure 2A), the enhanced formation of surface protrusions occurs in parallel with the rapid retraction of layers (at velocities too fast to track). At intermediate water content (Figure 2B), layers continue to retract at reduced rates, but still too fast to track by AFM. At the highest water content (Figure 2C), we observe few surface protrusions and the formation of numerous etch pits. Height profiles of these etch pits (Figure S4) reveal an average depth equivalent to one-half the unit cell in the *b*-direction of cholesterol hemiethanolate. Once an underlying layer is exposed within the etch pit, the newly generated steps retract as the surface dissolves. The shape of etch pits are irregular and do not mirror the morphology of bulk crystals, which is typical of etch pits reported for many systems.^53,54^ This suggests cholesterol crystals contain defects, which is consistent with previous single-crystal X-ray diffraction (SCXRD) data showing evidence of defects in both monohydrate and hemiethanolate crystals.^35^

**Figure 2:**
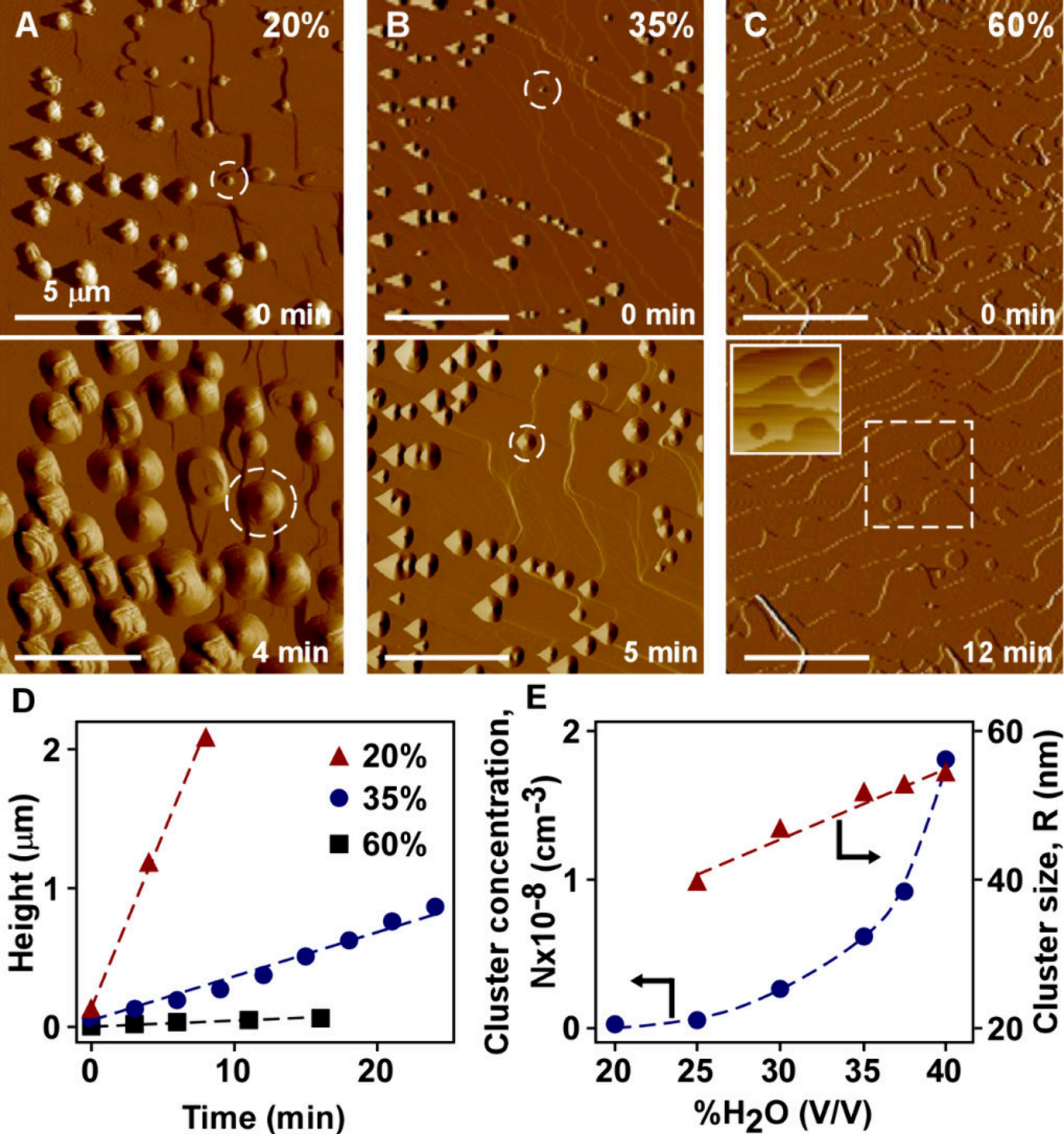
(A–D) Dissolution in cholesterol-free water/ethanol mixtures (C/C_eq_ = 0). AFM amplitude-mode images during the dissolution of cholesterol hemiethanolate (010) surfaces in an ethanol/water solvent with varying water content (by volume): (A) 20, (B) 35, and (C) 60% H_2_O. Images at initial times (top) and after continuous imaging for specified times (bottom) were obtained using a flow rate of 10 mL h^–1^. Inset of panel (C): height-mode image of etch pits within the highlighted region (dashed box). All scale bars equal 5 μm. (D) Change in height with imaging time for the protrusions highlighted by dashed circles in panels (A) and (B) (refer to Figure S3 for 60% water). (E) Comparison of cholesterol cluster concentration (circles, left *y*-axis) and size (triangles, right *y*-axis) in undersaturated ethanol/water bulk solutions (0.3 mM cholesterol) of varying water content. This plot was adapted from Chakraborty et al.^36^ Copyright 2025 American Chemical Society.

AFM measurements reveal that the height of the protrusions (Figure 2D) is a strong function of water content in the solvent. Time-resolved analysis of protrusion height shows a much higher rate of growth in solvents with less water compared to those with a majority of water where fewer protrusions are observed (Figure 2D). The protrusion height grows linearly with time for all solvent compositions, with a rate that dramatically drops from 200 to 4 nm min^–1^ as the water content increases from 20 to 60%, respectively. The exact composition and structure of the new phase formed at the crystal–solvent interface is unknown; however, we posit that it resembles solute-rich clusters that from in cholesterol solutions in water/ethanol mixtures.^36^ This conclusion is supported by the finding that clusters form in undersaturated solutions, whereas any other cholesterol crystal form, solvated or neat, would be expected to dissolve under similar conditions. We can also eliminate amorphous cholesterol as a possibility based on the fact that amorphous phases have higher solubility than crystalline phases.

Interestingly, at constant cholesterol concentration, the number of solute-rich clusters in bulk solution increases exponentially with increasing water content (Figure 2E), whereas the rate of growth of the protrusions decreases (Figure 2D). We attribute this discrepancy to the slower rate of dissolution at higher water content, which leads to reduced local concentration of free cholesterol near the crystal–solution interface and slower protrusion growth. One notable difference between the protrusions on crystal surfaces is a much larger size compared to the clusters formed in solution (Figure 2E). The fact that we do not see any apparent AFM tip effects on the size/shape of protrusions during continuous imaging seems to suggest they are not liquid, but likely solids or highly viscous gels.

The theory of microscopic reversibility for a reaction proposes that the forward and reverse processes occur with equal (but opposite) rates at equilibrium. Dissolution experiments were carried out in cholesterol-free solutions far from equilibrium (C_eq_); therefore, to test the limits of microscopic reversibility, we performed growth and dissolution experiments in a 35% (v/v) water/ethanol solvent using cholesterol concentrations above and below the equilibrium concentration, respectively. *In situ* AFM measurements were performed to obtain the step velocity, v_[100]_, in the [100] direction (Figure 3A) as a function of C/C_eq_, where *C* is the concentration of cholesterol with values of C/C_eq_ > 1 indicating supersaturated solutions, and values of C/C_eq_ < 1 designating undersaturated solutions. The response of v_[100]_ to changes in cholesterol concentration close to equilibrium is linear (Figure 3A, where the vertical dashed line indicates C/C_eq_ = 1). The constant slope of v_[100]_ versus C/C_eq_ in regions of growth and dissolution seems to suggest a reversible process that is consistent with time-resolved images of surfaces during growth (Figure S5) and dissolution (Figure 3B) where unfinished layers advance and retreat, respectively. The formation of etch pits (Figure 3B, arrows), however, clearly demonstrates a violation of microscopic reversibility at cholesterol concentrations close to solubility (i.e., as low as 5% undersaturation; Figure 3A, light blue shading). Once the degree of undersaturation reaches C/C_eq_ = 0.58, we observe the first evidence of protrusions forming on dissolving crystal surfaces (Figure 3C), thus signifying the shift to a nonclassical pathway that is even more divergent from microscopic reversibility. Beyond this point, there is a notable shift in v_[100]_ with decreasing C/C_eq_ (Figure 3A, dark blue shading) wherein protrusions reduce the velocity of step retraction and induce the formation of step bunches, which dissolve at slower rates than single layers.

**Figure 3:**
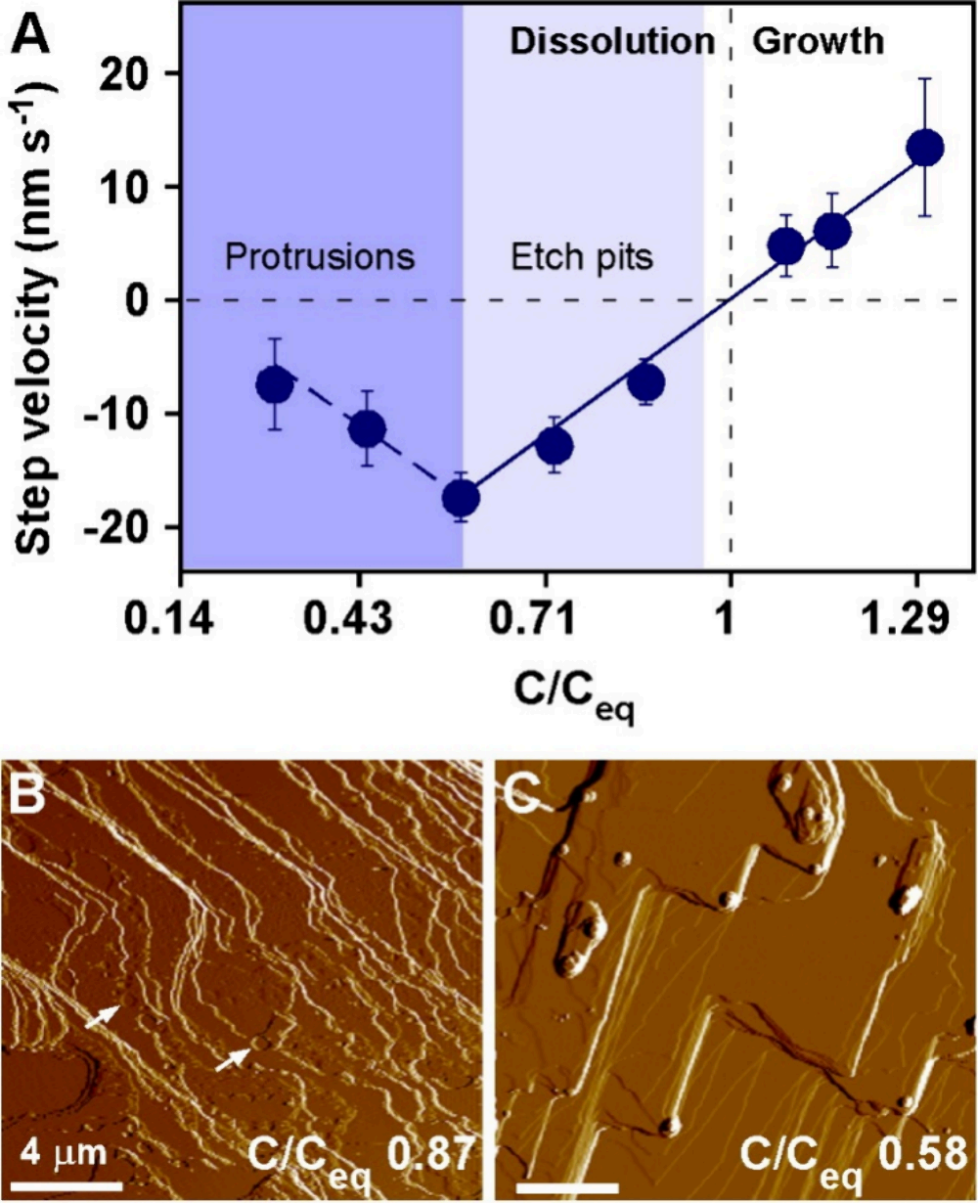
Limits of microscopic reversibility. (A) Step velocity v_[100]_ in the [100] direction for cholesterol hemiethanolate (010) surfaces in contact with 35% water/EtOH solutions at supersaturated (C/C_eq_ > 1) and undersaturated (C/C_eq_ < 1) conditions. Negative velocity (below the horizontal dashed line) corresponds to dissolution. Equilibrium (C/C_eq_ = 1) is denoted by the vertical dashed line. Data are the averages of 5 steps in a single measurement. Error bars span two standard deviations, and lines are linear regression. Light and dark shading highlight dissolution regimes violating microscopic reversibility via the formation of etch pits and protrusions, respectively. (B and C) Snapshots from Movies S2 and S3 of *in situ* AFM measurements at (B) C/C_eq_ = 0.87 mM and (C) C/C_eq_ = 0.58 mM, respectively. All AFM experiments were performed with a continuous flow of solution (30 mL h^–1^) selected at a volumetric rate to ensure growth and dissolution experiments were performed in a diffusion-limited regime. Arrows indicate etch pits. All scale bars equal 4 μm.

We also confirmed that the critical phenomena observed in Figure 3A for cholesterol hemiethanolate crystal dissolution in water/ethanol solvents is evident for cholesterol monohydrate crystal dissolution in water/IPA solvents. Notably, *in situ* AFM dissolution of cholesterol monohydrate crystals using a moderately undersaturated solution (C/*C*_*e*_ = 0.98 cholesterol in 50% H_2_O/IPA by volume) leads to etch pit formation (Figure S6). There is also an identical shift in dissolution mechanism to protrusion formation and growth at lower C/*C*_*e*_ ratios (Figure 1C–E). The similar behavior observed for pathologically relevant monohydrate crystals indicates microscopic reversibility is violated in both systems, further emphasizing the potential physiological significance of these processes.

### Proposed Mechanism of Heterogeneous Phase Transformation

When crystals grow or dissolve there is a boundary layer (BL) that develops in close proximity to the surface, creating a gradient in solute composition from the crystal surface to the bulk solution (far from the crystal–solvent interface). The thickness of this layer depends on the solution flow velocity;^38,55,56^ therefore, for evidence of a boundary layer effect, we measured the cholesterol crystal growth rate as a function of solution flow rate through the AFM liquid cell.^35^ These measurements revealed that at around 50 mL h^–1^ the growth regime shifts from a diffusion-limited to reaction-limited.^35^ To test the hypothesis that the protrusions form owing to the accumulation of cholesterol in this boundary layer, we performed dissolution experiments within the diffusion-limited regime using volumetric flow rates in the range 10–30 mL h^–1^. At higher solution flow rates, the thickness of the boundary decreases with increased flow rate, which allows cholesterol to diffuse from the crystal surface to bulk solution faster and in this way lowers the cholesterol concentration near the surface.

*In situ* AFM observations in cholesterol-free solutions flowing at four different rates of 5 mL h^–1^ (Figure 4A), 10 mL h^–1^ (Figure 4B), 30 mL h^–1^ (Figure 4C), and 60 mL h^–1^ (Figure S7) show a reduced number of protrusions with increasing flow rate. Measurements of protrusion growth (Figure 4D) reveal only a marginal reduction with increasing flow rate. This observation suggests surface diffusion is a dominant pathway for cholesterol incorporation into protrusions. At a flow rate of 30 mL h^–1^ a discontinuity in the height versus time plot appears, which coincides with the observation of step bunches in close proximity to protrusions (Figure 4C). Step bunches exhibit slower rates of step retreat compared to single layers, which dissolve at faster rates with increasing solution flow rate. After reaching the reaction-limited regime (60 mL h^–1^) where the dissolution rate is significantly higher and independent of flow rate, the trend is reversed and we observe surfaces with numerous protrusions exhibiting more diverse sizes (Figure S7).

**Figure 4:**
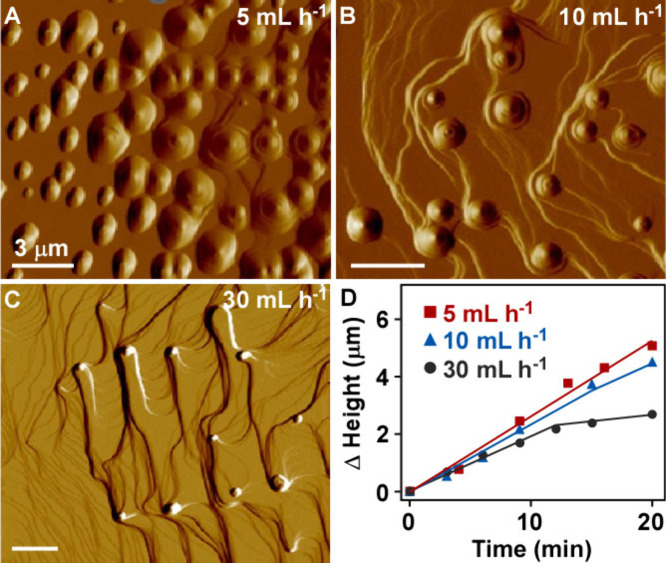
*In situ* AFM observations during cholesterol crystal dissolution showing the simultaneous growth of protrusions and retraction of underlying layers as a function of flow rate. Tapping-mode (amplitude) images of dissolving cholesterol hemiethanolate (010) surfaces show the changing topography with flow rates of (A) 5, (B) 10, and (C) 30 mL h^–1^ (Movies S4–S6, respectively). All measurements were performed in cholesterol-free solutions of 35% water/ethanol (v/v). (D) Corresponding evolution of protrusion height (scaled relative to time *t* = 0 min) with increased imaging time at each flow rate. Lines are interpolated to guide the eye.

The AFM observations at varying solution flow rates suggest three pathways along which protrusions form on cholesterol hemiethanolate surfaces in undersaturated media (Figure 5): (a) solid-state rearrangement without cholesterol being released into the boundary layer (Figure 5, *i*); (b) diffusion and aggregation of cholesterol monomers or oligomers released from dissolving crystal terraces (Figure 5, *ii*); and (c) formation of cholesterol-rich clusters within the boundary layer that attach to the crystal surface (Figure 5, *iii*). We also posit multiple pathways in which protrusions grow by surface diffusion (Figure 5, *iv*), direct attachment of monomers and/or oligomers from the boundary layer (Figure 5, *v*), or attachment of clusters from the BL (Figure 5, *vi*). AFM height profiles of growing protrusions (Figure S8) do not contain evidence of cluster attachment. Moreover, our previous study^36^ showed that cholesterol hemiethanolate grows classically from soluble species, but observed no evidence of nonclassical growth via the attachment of cholesterol-rich clusters. This implies the new phase grows via a process that violates microscopic reversibility, suggesting dissolution releases species of higher order than a monomer that are capable of generating cholesterol-solvate condensates as precursors for the formation and growth of surface protrusions.

**Figure 5:**
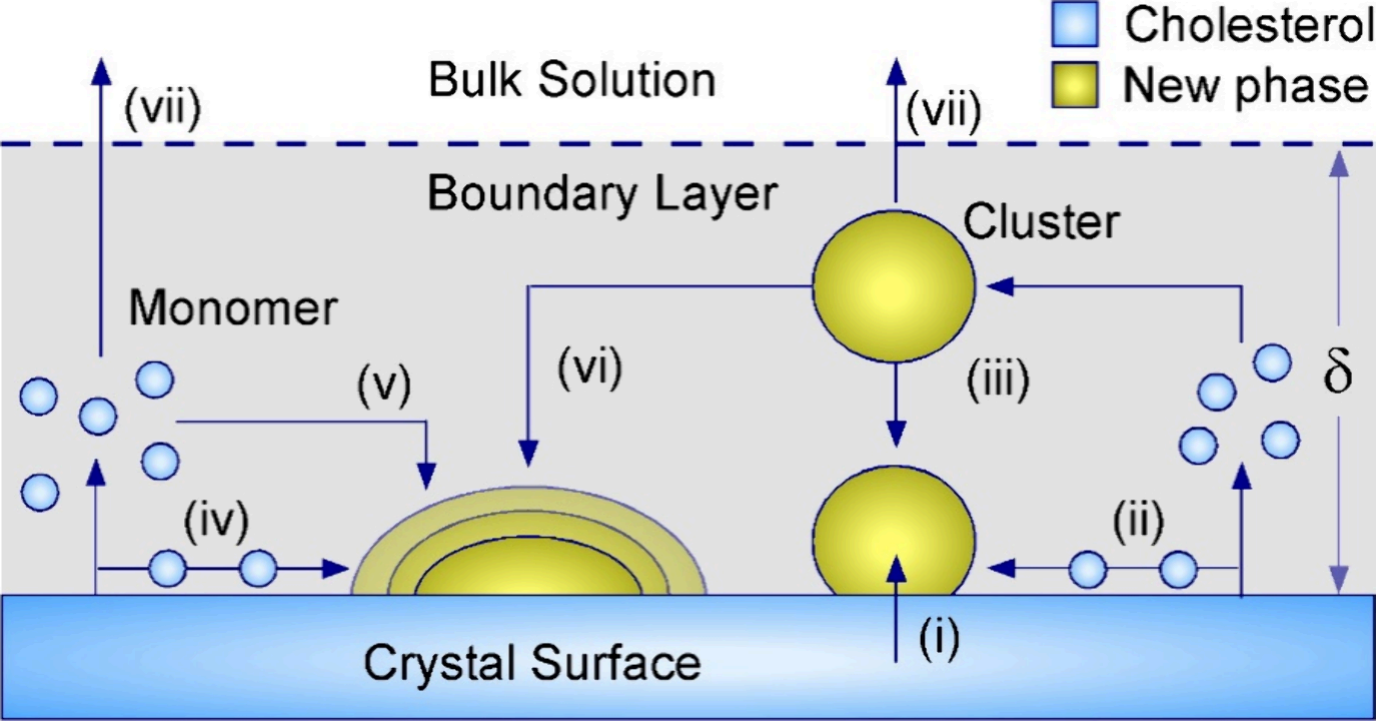
Idealized pathways of protrusion formation (i–iii) and growth (iv–vi) during the dissolution of cholesterol crystal surfaces in highly undersaturated solutions. The potential pathways for protrusion formation consist of (i) solid-state rearrangement, (ii) surface diffusion and aggregation of released monomers or oligomers, and (iii) attachment of cholesterol-rich clusters formed within the boundary layer (BL, dashed horizontal line). The putative pathways for protrusion growth consist of (iv) surface diffusion, (v) attachment of monomers/oligomers from the BL, and (vi) attachment of cholesterol-rich clusters from the BL. During dissolution, pathways (vii) allow for cholesterol monomers/oligomers and clusters within the BL to be released into bulk solution.

### Regeneration of Cholesterol Surfaces after Nonclassical Dissolution

Here we demonstrate that the new phase formed on surfaces of cholesterol crystals during periods of nonclassical dissolution impacts surface growth once crystals are placed in contact with a supersaturated medium. For these *in situ* AFM experiments we first assessed regeneration in the absence of the new phase wherein surfaces were first dissolved in a mildly undersaturated solution (*C*/*C*_*eq*_ = 0.95) that induces layer retraction and etch pit formation (Figure 6A), as well as a relatively high percentage of step bunches. After a period of dissolution the inlet solution to the AFM liquid cell was replaced with a supersaturated solution (*C*/*C*_*eq*_ = 1.27) and we observed immediate layer advancement (Figure 6B,C). A second experiment was conducted in which the surface was dissolved in a purely undersaturated solution (35% water/EtOH (v/v) with *C*/*C*_*eq*_ = 0), which leads to many protrusions (Figure 6D). The solution was then replaced with one having the same supersaturation as the previous experiment. Under these conditions, we observe much slower rates of growth for all unfinished layers in the vicinity of protrusions (Figure 6E,F). High magnification of the step edges in contact with protrusions (Figure 6D–F, insets) reveals that the steps are pinned, analogous to the action of foreign compounds employing a common inhibition mechanism (Figure 6G). The pinned steps bend in an attempt to penetrate the space between two pinners and the imposed curvature increases the chemical potential of the solute in the curved segment. The resulting decrease in supersaturation, which drives step growth, decreases the rate of layer advancement and the driving force for surface growth.^57–60^

**Figure 6:**
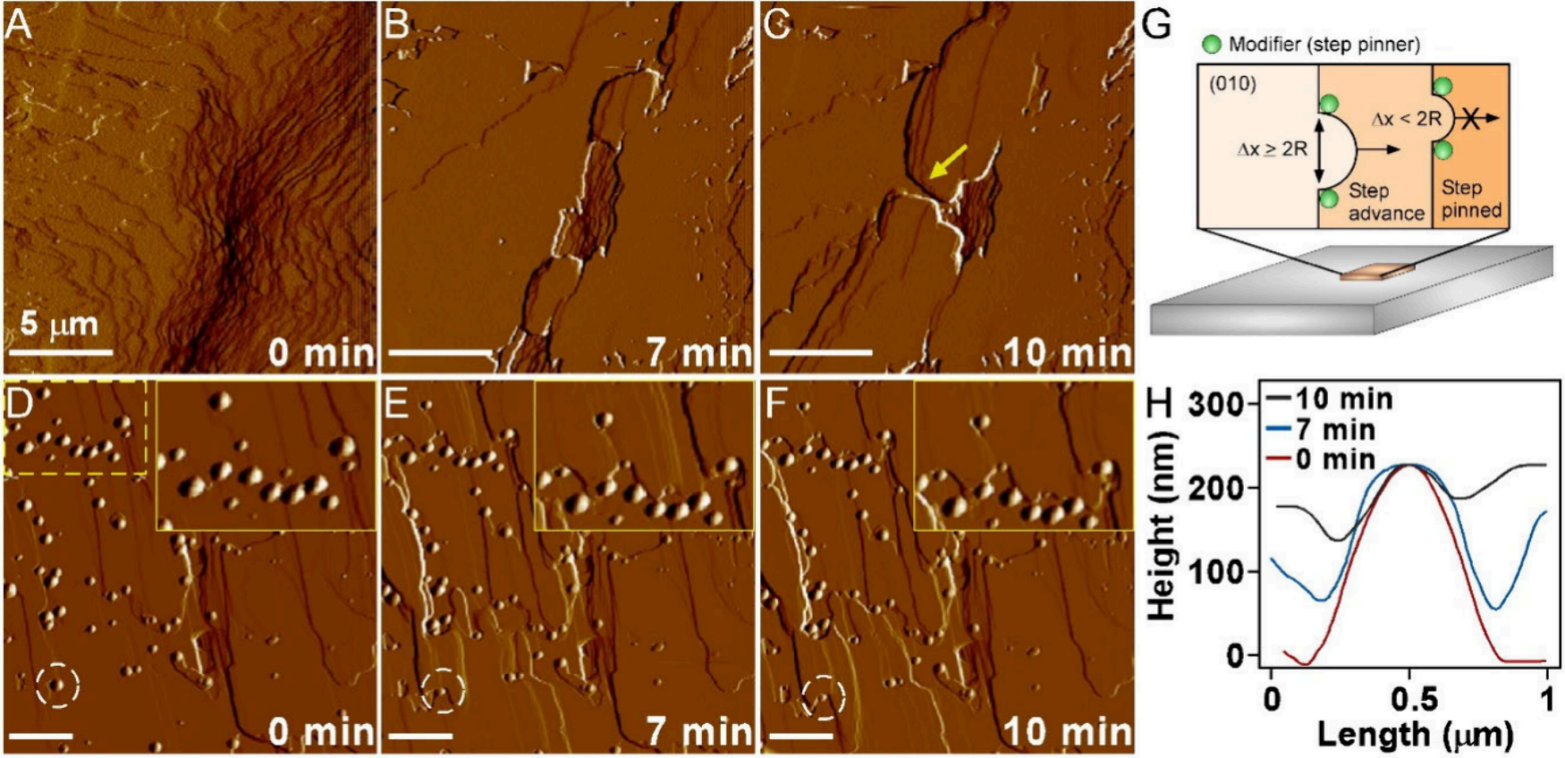
Regeneration of surface growth after dissolution. (A) AFM image of a cholesterol hemiethanolate (010) surface after less than 10 min of dissolution in a slightly undersaturated solution (*C*/*C*_*eq*_ = 0.95, 35% (v/v) water/EtOH mixture). (B and C) Regeneration of the surface in panel (A) by introducing a supersaturated solution (*C*/*C*_*eq*_ = 1.27) into the AFM liquid cell and monitoring growth over a 10 min period. (D) AFM image of a cholesterol hemiethanolate (010) surface after less than 10 min of dissolution in a purely undersaturated solution (*C*/*C*_*eq*_ = 0, 35% (v/v) water/EtOH mixture). (E and F) Regeneration of the surface in panel (D) by introducing a supersaturated solution (*C*/*C*_*eq*_ = 1.27) into the AFM liquid cell and monitoring growth over a 10 min period. Insets: yellow boxes are enlarged images of the region in the dashed box, highlighting an advancing step in contact with multiple protrusions. (G) Idealized scheme of a step-pinning mechanism of surface growth inhibition (R = critical radius of step curvature). (H) Time-resolved height profile of the protrusion in panels (D–F) highlighted by the dashed white circle. The profiles are shifted to align the maximum height of the protrusion.

The fate of protrusions during regeneration is difficult to resolve by *in situ* AFM imaging. As shown in Figure 6H, time-resolved changes in the height profile of a protrusion during regenerated growth of the (010) surface cannot determine if the new phase integrates into the crystal (i.e., undergoes a disorder-to-order transition). Likewise, the apparent reduction in protrusion height cannot be definitively associated with either its dissolution or occlusion within the growing crystal (as a defect) since the advancement of proximal layers leads to a progressively increasing baseline. We performed another experiment involving three stages: (i) dissolution at *C*/*C*_*eq*_ = 0; (ii) regenerative growth at *C*/*C*_*eq*_ = 1.27; and (iii) dissolution in slightly undersaturated solution (*C*/*C*_*eq*_ = 0.8). The last stage induced classical layer dissolution, but also resulted in the dissolution of protrusions (Figure S9). The presence of protrusions also hindered layer retraction, resulting in a series of dependent processes that were difficult to deconvolute.

It should be noted that the effects of protrusions on surface growth in supersaturated solutions appear to last a limited time. At the supersaturation used in Figure 6 (*C*/*C*_*eq*_ = 1.27) to assess regeneration, layer growth is slow. In order to monitor the dynamics of regeneration at a reasonable time scale, we accelerated surface growth by increasing cholesterol supersaturation (e.g., *C*/*C*_*eq*_ = 1.31) where we observed overgrowth of surface protrusions within 60 min (Figure S10). Time-resolved AFM images seemingly show protrusions occluded within the crystal, which can impose strain. Movie S7 also shows regions composed of macrosteps that are frozen throughout the entire duration of AFM imaging, confirming that regeneration is incomplete. At significantly longer times (beyond our capability to monitor by AFM), it may be possible that surface overgrowth will eventually lead to a (010) surface that resembles the initial crystal prior to dissolution; however, for the purposes of our experiments we refer to the effects imposed by extreme dissolution leading to the generation of protrusions to be an irreversible phenomenon.

Bulk crystallization experiments reveal that the formation of protrusions during nonclassical dissolution occurs on the entire cholesterol hemiethanolate (010) surface, and not just in local regions of AFM imaging. Optical micrographs of crystals before (Figure 7A) and after (Figure 7B) dissolution clearly show a shift in translucent properties. At higher magnification (Figure 7C and D, respectively), there is clear evidence of darker spheres spread throughout the surface of a partially dissolved crystal. The ability of these protrusions to function as native inhibitors of surface growth during a regeneration period has potential implications for a “self-inhibition” mechanism in physiological environments, leading to potential growth cessation. We confirmed for cholesterol monohydrate that protrusions formed after partial dissolution inhibit layer advancement during attempts to regenerate surface growth (Figure S11). If cholesterol crystals in the human body reside in regions where soluble cholesterol levels cycle between undersaturated and saturated conditions, there could be an irreversible effect on cholesterol crystallization similar to the observations reported here. The media used in this study, however, are far from physiological; and thus, the hypothesis of self-inhibition remains an open subject for future investigation.

**Figure 7:**
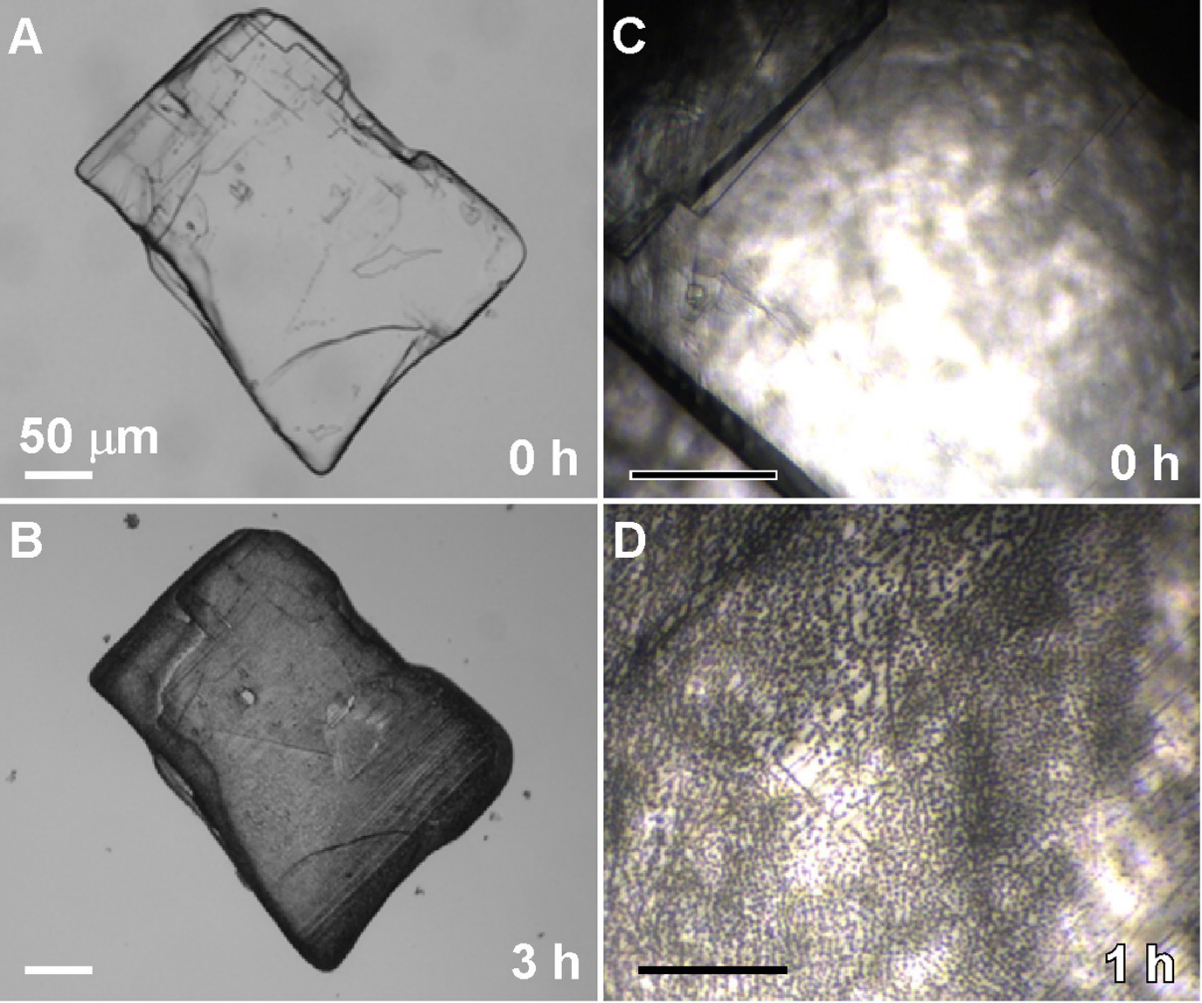
Macroscopic evidence of nonclassical dissolution. (A and B) Optical micrographs of a cholesterol hemiethanolate crystal prepared in bulk using a 35% (v/v) water/EtOH mixture (panel (A)) and then dissolved in pure solvent for 3 h (panel (B)). (C and D) Optical micrographs during an *in situ* AFM experiment showing the surface before (panel (C)) and after (panel (D)) dissolution for 1 h, where we observe the emergence of surface protrusions (i.e., dark circles). All scale bars equal 50 μm.

## Conclusions

In summary, we explored the dissolution mechanism of both monohydrate and hemiethanolate cholesterol crystals with the objective to establish a platform for identifying molecular processes that modify or suppress crystal dissolution. In addition to the expected retracting crystal layers, several structures appear on dissolving crystal surfaces in undersaturated media: etch pits, step bunches, and, most unexpectedly, protrusions. We established that the protrusions represent cholesterol solid aggregates that form by material released during crystal dissolution and are akin to the cholesterol-rich clusters that exist in low-concentration cholesterol solutions. The protrusions slow down the advancement of retracting steps and, in this way, violate the symmetry between growth and dissolution, a manifestation of the principle of microscopic reversibility. They interfere with the collective behaviors of retracting crystal layers and enhance step bunching. We find that the protrusions and step bunches play a major role during surface regeneration in supersaturated solutions wherein they act as self-generated inhibitors that pin and bend the steps growing around them and promote bunching of steps during growth.

Our findings show that even at 5% undersaturation, the formation of etch pits during cholesterol crystal dissolution violates microscopic reversibility; however, the latter can easily be misinterpreted by the linear relationship between step velocity and cholesterol concentration, which persists until 42% undersaturation where dissolution shifts to the nonclassical mechanism. This unique mode of dissolution involving the formation of surface protrusions is uncommon, and may have potential implications for physiological processes that occur when local environments cycle between stages of supersaturation and undersaturation. Moreover, the dynamics of the collective behaviors of cholesterol molecules during the retraction and growth of steps provide a foundation for the identification of modifiers that might regulate etch pits, protrusions, and step bunches toward the design of a strategy to destabilize cholesterol-crystal containing arterial plaques and gallstones.

## Supplementary Material

















## Notes

Experimental methods, powder X-ray diffraction patterns from cholesterol crystal stability tests, additional atomic force microscopy images from *in situ* measurements at various conditions, and full descriptions of supporting movies (PDF)
Movie S1: Dissolution of surface layers and protrusion growth in a completely undersaturated solution (C = 0 mM) containing 35% (v/v) water/IPA mixture at a flow rate of 10 mL h^–1^ (imaging area is 27 μm × 26 μm) (MP4)
Movie S2: Surface dissolution mechanism in a slightly undersaturated solution (C/C_eq_ = 0.87) without forming any protrusions on the surface containing 35% (v/v) water/EtOH solvent at a flow rate of 30 mL h^–1^ (imaging area is 17 μm × 16 μm) (MP4)
Movie S3: Surface dissolution mechanism in a moderately undersaturated solution (C/C_eq_ = 0.58) while protrusions start to form on the surface containing 35% (v/v) water/EtOH solvent at a flow rate of 30 mL h^–1^ (imaging area is 17 μm × 16 μm) (MP4)
Movie S4: Surface dissolution mechanism in a purely undersaturated solution (C/C_eq_ = 0) at a flow rate of 5 mL h^–1^ showing many protrusions with higher growth rate on the surface containing 35% (v/v) water/EtOH solvent (imaging area is 16 μm × 13 μm) (MP4)
Movie S5: Surface dissolution mechanism in a purely undersaturated solution (C/C_eq_ = 0) at a flow rate of 10 mL h^–1^ showing many protrusions with higher growth rate on the surface containing 35% (v/v) water/EtOH solvent (imaging area is 15 μm × 12 μm) (MP4)
Movie S6: Surface dissolution mechanism in a purely undersaturated solution (C/C_eq_ = 0) at a flow rate of 30 mL h^–1^ showing many protrusions with higher growth rate on the surface containing 35% (v/v) water/EtOH solvent (imaging area is 23 μm × 19 μm) (MP4)
Movie S7: Overgrowth of protrusions by layer growth and regions of incomplete regeneration in a supersaturated solution (C/C_eq_ = 1.31) at a flow rate of 60 mL h^–1^ (imaging area is 20 μm × 20 μm) (MP4)
