# Supplementary material for: Cholesterol Crystallization Tests the Limits of Microscopic Reversibility for Molecular Pathways of Growth and Dissolution
Source: ACS Cent Sci. 2026 Apr 24;12(5):616–26. doi: 10.1021/acscentsci.5c02371 (PMC13220209; doi:10.1021/acscentsci.5c02371)
Supplement: Supplementary file 1 [file oc5c02371_si_001.pdf]

Supporting Information for

Cholesterol Crystallization Tests the Limits of Microscopic Reversibility for Molecular Pathways of Growth and Dissolution

Dipayan Chakraborty[1,2], Muhammad Osman Khalid[1,2], Peter G. Vekilov[1,2,3], and Jeffrey D. Rimer[1,2,3,*]

[1] *William A. Brookshire Department of Chemical and Biomolecular Engineering, University of Houston, 4226 Martin Luther King Blvd., Houston, TX 77204-4004, USA*
[2] *Welch Center for Advanced Bioactive Materials Crystallization*
[3] *Department of Chemistry, University of Houston, 3585 Cullen Blvd., Houston, TX 77204-5003, USA*
*Correspondence sent to: jrimer@central.uh.edu

Table of Contents **Page**

Experimental Methods………………………………………………………………… S2
Supporting Figures……………………………………………………………………….. S3
Movies…………………………………………………………………………………... S10

List of Supporting Figures

Figure S1: Stability analysis of cholesterol crystals in different solvents and air.
Figure S2: AFM snapshots of cholesterol hemiethanolate dissolution at 0 mM cholesterol.
Figure S3: Surface dynamics of cholesterol hemiethanolate dissolution at varying water content.
Figure S4: Dissolution of cholesterol hemiethanolate involving etch pit formation.
Figure S5: AFM snapshots of cholesterol hemiethanolate dissolution at varying degrees of saturation.
Figure S6: Etch pit formation during cholesterol monohydrate dissolution.
Figure S7: AFM snapshot of cholesterol hemiethanolate dissolution at a flowrate of 60 mL h^{-1}.
Figure S8: Time-resolved analysis of protrusion growth during cholesterol hemiethanolate dissolution.
Figure S9: Role of protrusions on cholesterol hemiethanolate surfaces during dissolution and growth.
Figure S10: Regeneration of cholesterol hemiethanolate (010) surface after partial dissolution.
Figure S11: Regeneration of cholesterol monohydrate (001) surface in the presence of protrusions.

Experimental Methods

Materials. The following compounds were purchased from Sigma Aldrich (St. Louis, MO): anhydrous cholesterol powder (≥ 99%), ethanol (EtOH, ≥ 99.5%), and isopropanol (IPA, ≥ 99.5%). Deionized (DI) water was produced by an Aqua Solutions RODI water purification system (18.2 MΩ).

Bulk cholesterol crystallization. Cholesterol crystals were prepared by three different methods: (i) slow cooling of a supersaturated solution that was first heated to dissolve cholesterol; (ii) incubation of anhydrous cholesterol powder in solution; and (iii) slow evaporation at room temperature of an initially undersaturated solution. In the first method, anhydrous cholesterol powder was added to either a water/IPA or water/EtOH mixture and the resulting mixture was heated at 45°C until all cholesterol was fully dissolved. Supersaturation was induced by cooling these solutions at a rate of 4°C min^{-1} until reaching room temperature. Both solvents produced crystals with large platelet morphologies. Crystals prepared in water/IPA mixtures were removed from solution by filtration (Whatman, 0.22 μm) and stored under air at ambient conditions prior to powder X-ray diffraction (PXRD) measurements using a Rigaku diffractometer with Cu Kα radiation (40 kV, 40 mA). Samples for PXRD were prepared by grinding with a mortar and pestle. The same procedure was repeated for crystals prepared in water/EtOH mixtures, but without storage to minimize EtOH evaporation from cholesterol hemiethanolate crystals. In the second method, anhydrous cholesterol powder was incubated in pure solvents (ethanol or DI water) to form pure solvated/hydrated structures. In the third method, anhydrous cholesterol powder was dissolved based on its solubility at room temperature and the solution was then evaporated through holes placed on the top of the glass vial. During evaporation, the supersaturation increased gradually, leading to large crystals (>500 μm) used as substrates for atomic force microscopy (AFM) experiments. *In situ* monitoring of bulk cholesterol crystal dissolution was performed by first preparing crystals as described above in a 20 mL glass vial and then transferring them to another vial with purely undersaturated solutions. Dissolution of these crystals was monitored with an inverted optical microscope (Leica DMi8 instrument).

AFM measurements of cholesterol crystal surface growth and dissolution. Cholesterol crystals prepared by the slow evaporation method were used for *in situ* AFM measurements. Crystals were taken out of the solution and adhered on an AFM specimen disk (Ted Pella Inc.) using clear quickset Gorilla epoxy. A Multimode 8 Bruker AFM was used in tapping mode for all experiments in this study. Images were collected over 20 min of continuous measurements using a ScanAsyst-Fluid probe (silicon nitride, 150 kHz, 0.7 N/m). Cholesterol solutions prepared with either a supersaturated or undersatured condition for growth or dissolution, respectively, were continuously supplied to the AFM liquid cell using a syringe pump (CHEMYX Fusion 101A) at a fixed volumetric flow rate.

Supporting Figures

Stability analysis of cholesterol crystals. We studied the stability of cholesterol crystals in air and different solvents using two different methods to prepare cholesterol crystals: (i) incubating pure cholesterol crystals in different solvents; and (ii) crystallizing cholesterol crystals from supersaturated solutions by slow cooling. In the first method, crystals were prepared by incubating anhydrous cholesterol powder in different solvents. Incubating anhydrous crystal in pure ethanol forms pure hemiethanolate (characteristic peak *b* in Figure S1A) whereas in pure water leads to monohydrate formation (characteristic peak *c* in Figure S1A). These crystals undergo solvate/hydrate transformation while incubated in respective pure solvents (ethanol or water). This confirms that these crystalline forms have high dependencies on the solvent. In the second method, crystals were prepared by making supersaturated solutions based on the equilibrium concentrations at 45°C and cooling it down to room temperature at a rate of 4°C h^{-1}. Plate-like crystals formed in different solvents as hemiethanolate (monoclinic) and monohydrate (triclinic) have similar morphology. These crystals were taken out of the solution kept in air to check their stability. It was observed that the PXRD peak (labelled *b* in Figure S1B) of hemiethanolate crystals prepared in 50%H_2O/EtOH is reduced while the peak of monohydrate (labelled *c* in Figure S1B) remains the same whereas the anhydrous peak (labelled *a* in Figure S1B) increases. This suggests that hemiethanolate loses ethanol from the crystal structure and becomes anhydrous while monohydrate crystals remain stable.

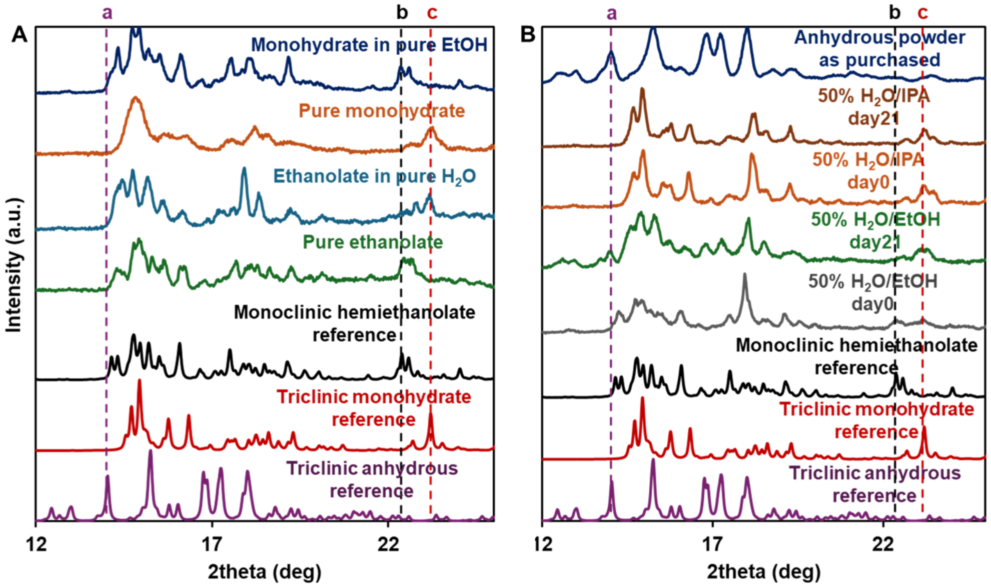

Figure S1. Assessment of cholesterol monohydrate (triclinic) and cholesterol hemiethanolate (monoclinic) crystal stability in (A) different solvents and (B) air at ambient conditions. (A) Powder X-ray diffraction (XRD) patterns of solids placed in different solutions at room temperature for at least 7 days: (i) Monohydrate crystals placed in pure EtOH undergo a transition to hemiethanolate crystals; (ii) anhydrous cholesterol (as-received reagent) placed in pure DI water changes to monohydrate crystals; (iii) Hemiethanolate crystals placed in pure DI water undergo a transition to monohydrate crystals; (iv) anhydrous cholesterol (as-received reagent) placed in pure EtOH changes to hemiethanolate crystals; (v – vii) Reference patterns obtained from the Cambridge Crystallographic Data Center (CCDC) for monoclinic cholesterol hemiethanolate (CHOLEU10), triclinic cholesterol monohydrate (CHOLES20), and triclinic anhydrous cholesterol (CHOEST10) where the vertical dashed lines indicate characteristic peaks for each structure (labelled *b*, *c*, and *a*, respectively). (B) Powder XRD patterns for the following samples: (i) anhydrous cholesterol (as-received reagent); (ii and iii) Monohydrate crystals prepared from a solution of 50/50 IPA/H_2O that were placed in air for 0 and 21 days; (iv and v) Hemiethanolate crystals prepared from a solution of 50/50 EtOH/H_2O that were placed in air for 0 and 21 days; (vi – viii) Identical reference patterns as those in panel A.

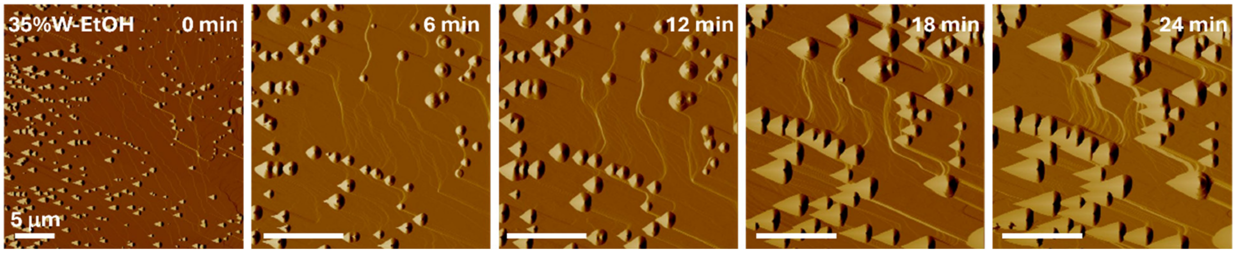

Figure S2. *In situ* AFM measurements during dissolution of a cholesterol hemiethanolate (010) surface in a purely undersaturated (0 mM cholesterol, S = 0) solution of 35% (v/v) water/ethanol. Images were taken over a 24-min period of continuous measurements using a fixed volumetric flow rate (10 mL h^{-1}) of solution into the liquid cell. All scale bars equal 5 µm.

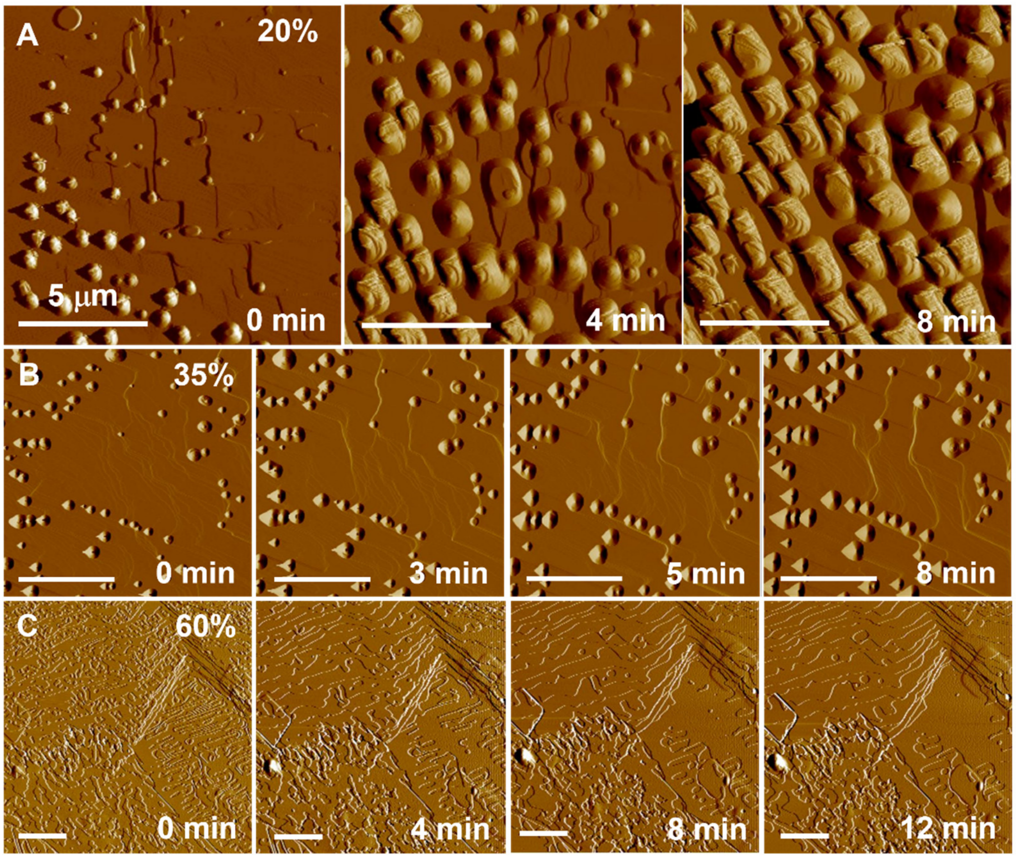

Figure S3. Time-resolved AFM measurements during dissolution of a cholesterol hemiethanolate (010) surface in a purely undersaturated (0 mM cholesterol, S = 0) solution of water/ethanol (v/v) mixtures with the following water contents; (A) 20, (B) 35, and (C) 60%. Imaging times are listed and all scale bars equal 5 µm.

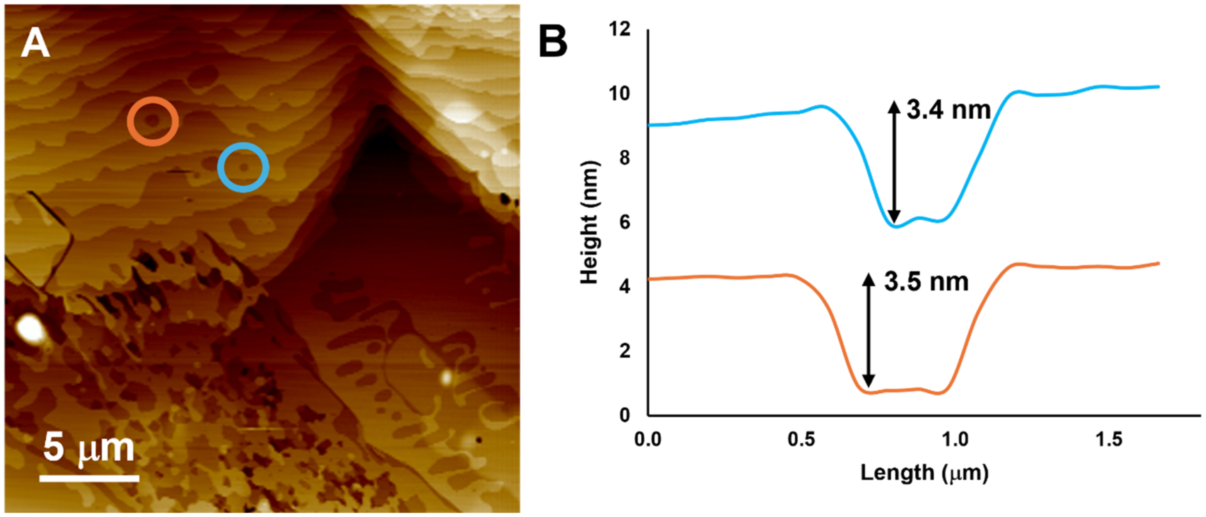

Figure S4. (A) Snapshot during an *in situ* AFM measurements of a cholesterol hemiethanolate (010) surface dissolving in a purely undersaturated (0 mM cholesterol, S = 0) solution of 60% (v/v) water/EtOH. (B) Height profile of two different etch pits (red and blue circles) with similar depth equal to one-half the unit cell dimension in the *b*-direction.

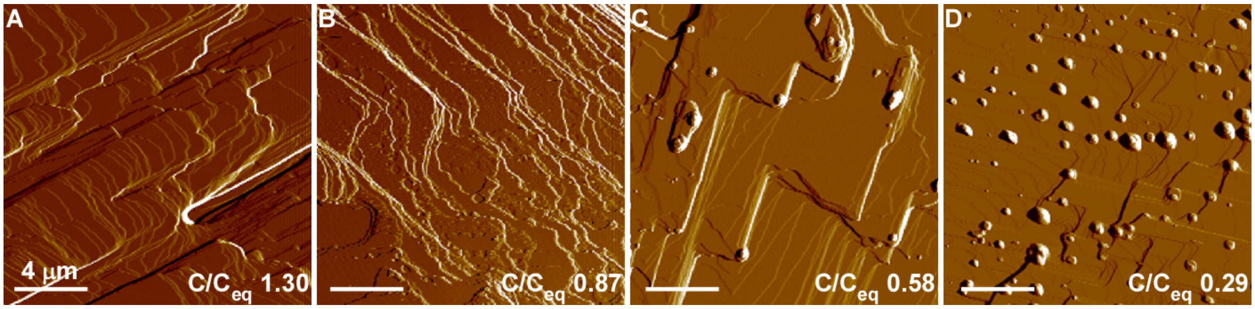

Figure S5. *In situ* AFM measurements of cholesterol hemiethanolate (010) surfaces in solutions of 35% (v/v) water/ethanol with the following cholesterol concentrations relative to its equilibrium concentration (C_{eq}): (A) C/C_{eq} = 1.30, (B) 0.87, (C) 0.58, and (D) 0.29.

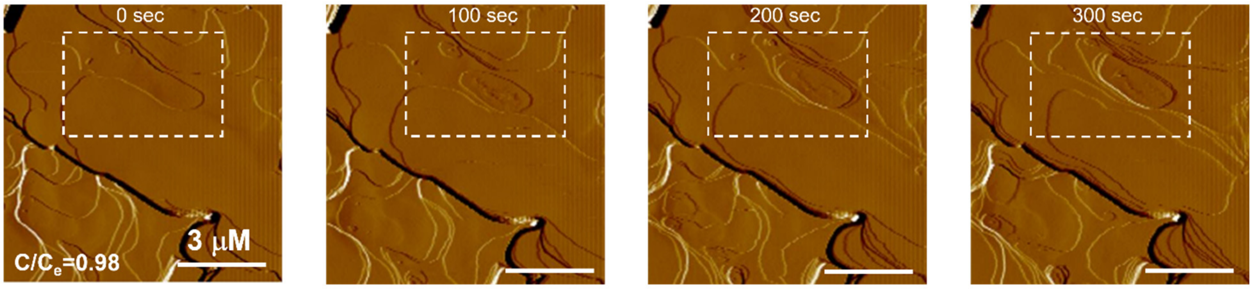

Figure S6. Time-resolved AFM measurements of a cholesterol monohydrate (001) surface dissolving in a mildly undersaturated (1.3 mM cholesterol, $C/C_{eq} = 0.98$) solution of water-IPA (50% water by volume) under continuous flow (60 mL h^{-1}) showing dissolution via etch pit formation (white dashed box).

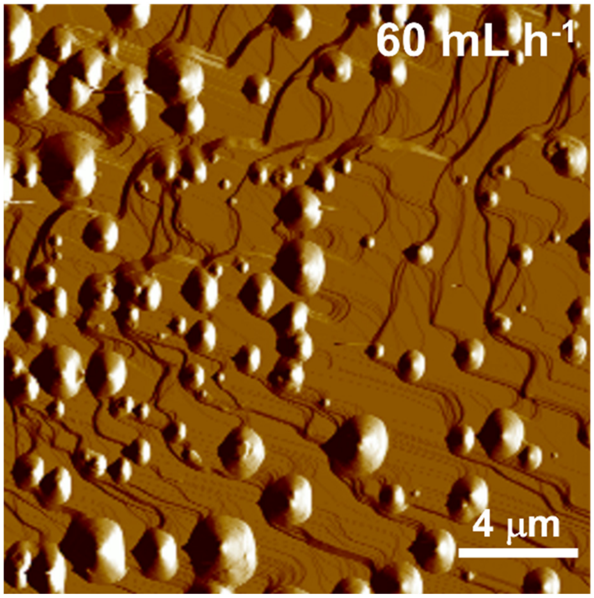

Figure S7. Snapshot during *in situ* AFM measurements of a cholesterol hemiethanolate (010) surface dissolving in a purely undersaturated (0 mM cholesterol, S = 0) solution of 35% (v/v) water/ethanol at a flow rate of 60 mL h^{-1}.

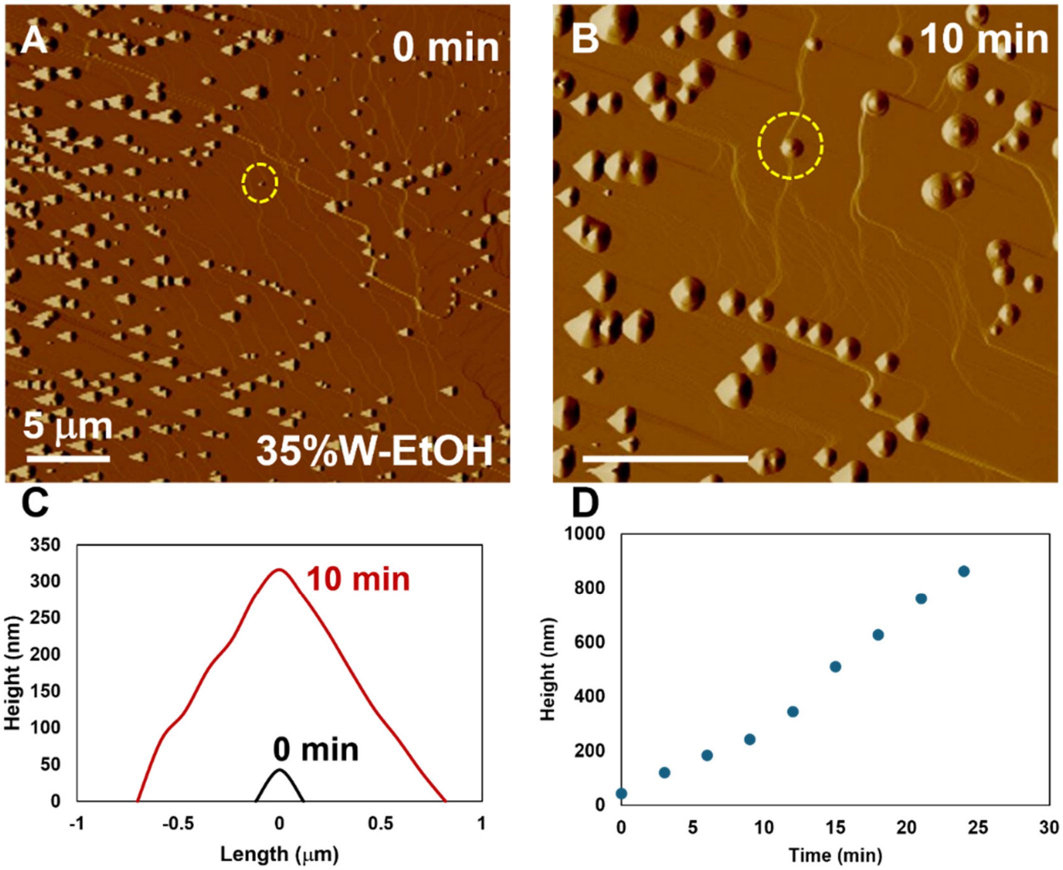

Figure S8. (A and B) *In situ* AFM measurements of a cholesterol hemiethanolate (010) surface dissolving over a 10-min period in a purely undersaturated (0 mM cholesterol, S = 0) solution of 35% (v/v) water/ethanol. (C) Height profile of the protrusion in panels A and B marked with the dashed yellow circle. (D) Temporal increase in the height of the same protrusion over a 25-min period of continuous crystal dissolution.

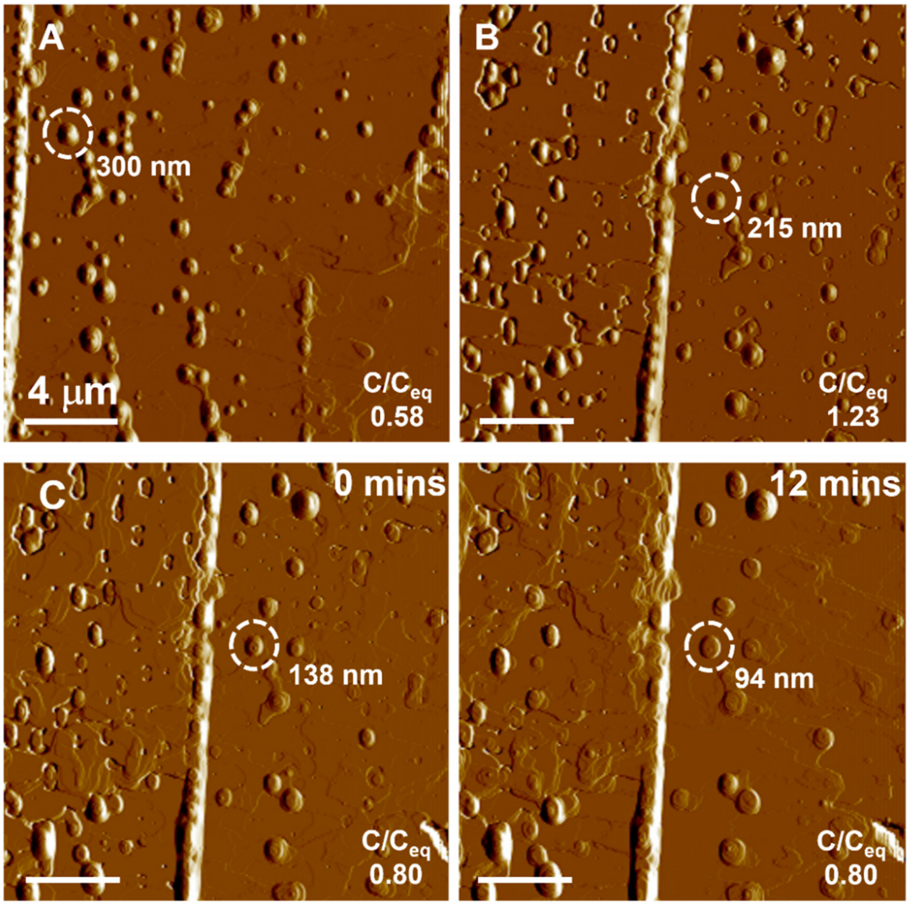

Figure S9. (A and B) *In situ* AFM measurements of a cholesterol hemiethanolate (010) surface (A) dissolving in undersaturated (0.4 mM cholesterol, S = 0.58) and (B) growing in supersaturated (0.85 mM cholesterol, S = 1.23) solutions of 35% (v/v) water/ethanol, respectively. (C) *In situ* AFM measurements of the same cholesterol hemiethanolate (010) surface as A and B dissolving over a 12-min period in a mildly undersaturated (0.55 mM cholesterol, S = 0.80) solution of 35% (v/v) water/ethanol. Dashed circle shows the same protrusion with its maximum height from the base for each measurement listed in nm.

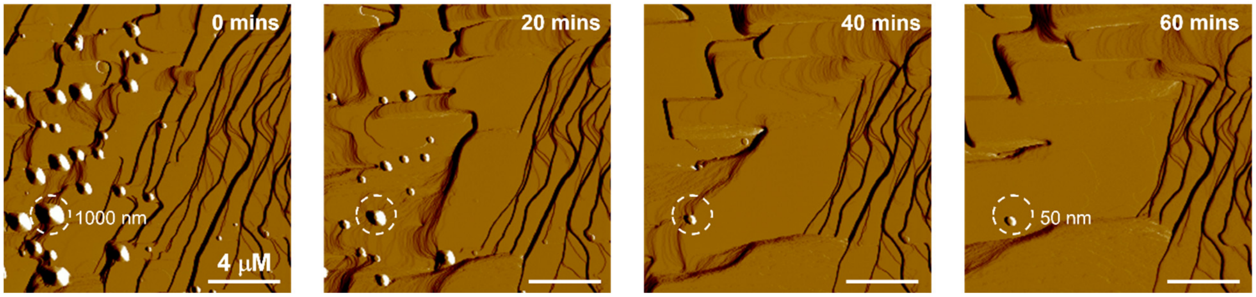

Figure S10. Time resolved AFM images of a cholesterol hemiethanolate (010) surface growing for one hour under continuous flow at a flowrate of 60 mL h^{-1} in a supersaturated solution (35% water-ethanol (v/v) with C/C_{eq} = 1.31) after partial dissolution in a purely undersaturated (C/C_{eq} = 0) water-ethanol mixture (35% water by volume) to obtain protrusions. White dashed circle shows a single protrusion with its maximum height from the surface listed as the protrusions are overgrown by advancing layers.

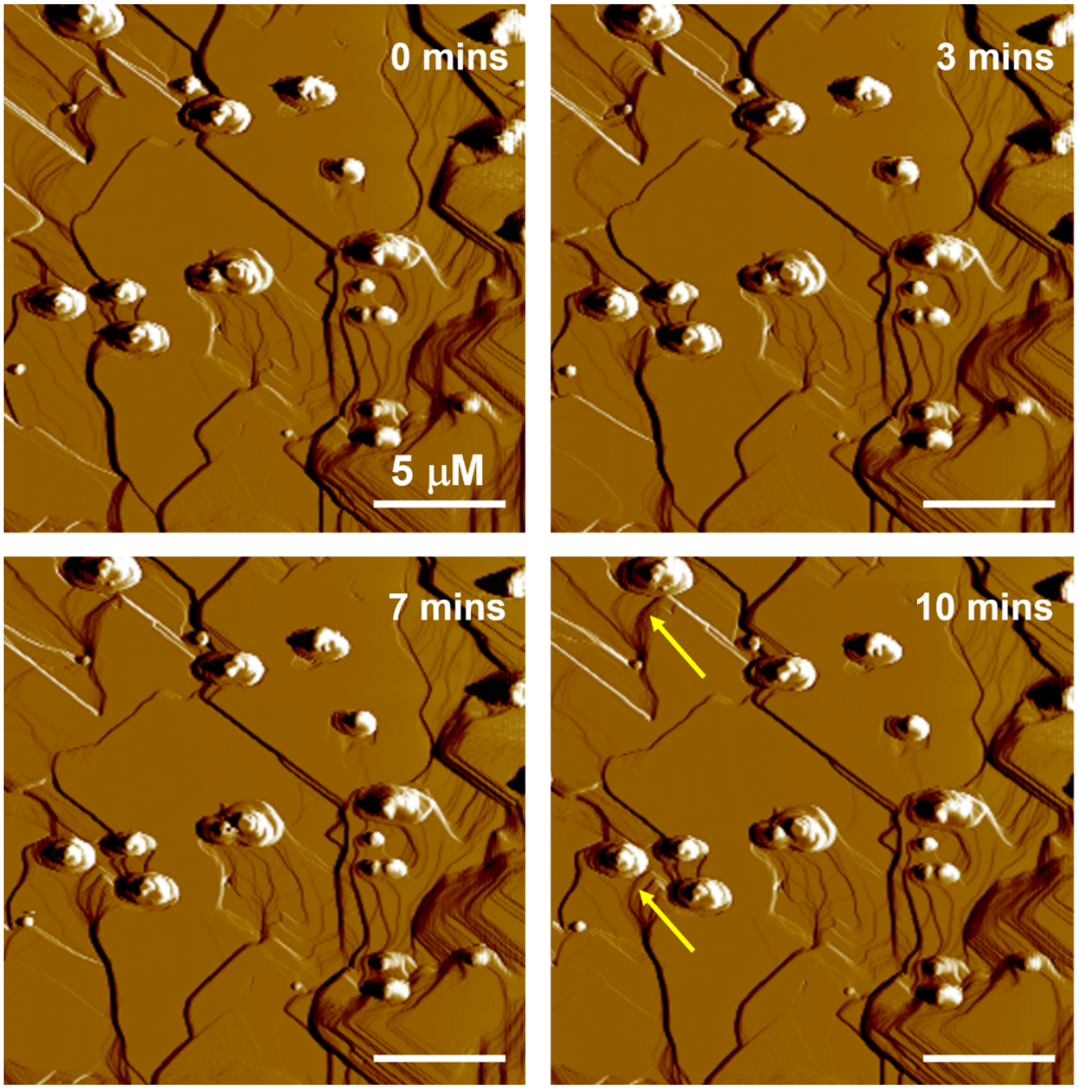

Figure S11. Time resolved AFM images of a cholesterol monohydrate (001) surface growing under continuous flow (60 mL h^{-1}) in a supersaturated solution (50% water-IPA (v/v) with C/C_{eq} = 1.06) after partial dissolution in a purely undersaturated (C/C_{eq} = 0) water-IPA mixture (35% water by volume) to obtain protrusions. Steps are stagnant near regions with high protrusion density. Yellow arrows show protrusions affecting layer advancement by causing steps to grow around them.

Supporting Movies

Movie S1. Dynamics of cholesterol monohydrate (001) crystal surface dissolution during *in situ* AFM experiments. The video reveals dissolution of surface layers and protrusion growth in a completely undersaturated solution (C = 0 mM) containing 35% (v/v) water/IPA mixture at a flowrate of 10 mL h^{-1}. The imaging area is 27 μm x 26 μm.

Movie S2. Dynamics of cholesterol hemiethanolate (010) crystal surface dissolution during *in situ* AFM experiments. The video reveals surface dissolution mechanism in a slightly undersaturated solution (C/C_{eq} = 0.87) without forming any protrusions on the surface containing 35% (v/v) water/EtOH solvent at a flowrate of 30 mL h^{-1}. The imaging area is 17 μm x 16 μm.

Movie S3. Dynamics of cholesterol hemiethanolate (010) crystal surface dissolution during *in situ* AFM experiments. The video reveals surface dissolution mechanism in a moderately undersaturated solution (C/C_{eq} = 0.58) while protrusions start to form on the surface containing 35% (v/v) water/EtOH solvent at a flowrate of 30 mL h^{-1}. This experiment tests the limit of microscopic reversibility on a cholesterol hemiethanolate surface in the undersaturated regime. The imaging area is 17 μm x 16 μm.

Movie S4. Dynamics of cholesterol hemiethanolate (010) crystal surface dissolution during *in situ* AFM experiments. The video reveals surface dissolution mechanism in a purely undersaturated solution (C/C_{eq} = 0) at a flowrate of 5 mL h^{-1} showing many protrusions with higher growth rate on the surface containing 35% (v/v) water/EtOH solvent. The imaging area is 16 μm x 13 μm.

Movie S5. Dynamics of cholesterol hemiethanolate (010) crystal surface dissolution during *in situ* AFM experiments. The video reveals surface dissolution mechanism in a purely undersaturated solution (C/C_{eq} = 0) at a flowrate of 10 mL h^{-1} showing many protrusions with higher growth rate on the surface containing 35% (v/v) water/EtOH solvent. The imaging area is 15 μm x 12 μm.

Movie S6. Dynamics of cholesterol hemiethanolate (010) crystal surface dissolution during *in situ* AFM experiments. The video reveals surface dissolution mechanism in a purely undersaturated solution (C/C_{eq} = 0) at a flowrate of 30 mL h^{-1} showing many protrusions with higher growth rate on the surface containing 35% (v/v) water/EtOH solvent. The imaging area is 23 μm x 19 μm.

Movie S7. Dynamics of cholesterol hemiethanolate (010) crystal surface growth regeneration after partial dissolution during *in situ* AFM experiments. The video reveals the overgrowth of protrusions by layer growth and regions of incomplete regeneration in a supersaturated solution (C/C_{eq} = 1.31) at a flowrate of 60 mL h^{-1}. The imaging area is 20 μm x 20 μm.
